# Descriptions of Immatures of the South American Plant Hopper, *Taosa* (*C*.) *longula*

**DOI:** 10.1673/031.012.14201

**Published:** 2012-12-06

**Authors:** Ana M. Marino de Remes Lenicov, María C. Hernández, María E. Brentassi, Bárbara Defea

**Affiliations:** ^1^ Universidad Nacional de La Plata, División Entomología., Facultad de Ciencias Naturales y Museo. Paseo del Bosque s/n. (1900), La Plata, Argentina.; ^2^ USDA-ARS-South American Biological Control Laboratory. (B1686EFA) Hurlingham, Argentina

**Keywords:** Argentina, biology, morphology, nymphal development, Perú, planthoppers

## Abstract

Descriptions of the immature stages of *Taosa* (*Cuernavaca*) *longula* Remes Lenicov (Hemiptera: Fulgoroidea: Dictyopharidae) and a key for their identification is provided for specimens collected on the water hyacinth, *Eichhornia crassipes* (Martius) Solms-Laubach (Commelinales: Pontederiaceae), in northeastern Argentina and Peru. Newly emerged nymphs from eggs collected in the field were reared in rearing chambers, and each stage was fixed to microscopic examination and illustration. Fifth nymphal instars can be easily recognized from congeners by the brown marked pattern coloration, shorter vertex, and the distinguishable median carina along the frons. Information on behavior and developmental time is also included.

## Introduction

*Taosa* (*Cuernavaca*) *longula* Remes Lenicov (Hemiptera: Fulgoroidea: Dictyopharidae) was recently described as a new species (Remes Lenicov and Hernández 2010) and as a part of a group of three sibling species, including *T. inexacta* (Walker) and one undescribed *Taosa* sp. The three species are sympatric in some South American tropical regions, as inferred from collection sites recorded by the second author and the *T. inexacta* geographic distribution from Bennet and Zwolfer (1968). Consequently, it is important to be able to accurately identify the three species in order to conduct studies on their life history and potential economic importance.

Information on life history is only available for a few species of dictyopharids (Wilson and McPherson 1981; Wilson and Wheeler 1992; Emeljanov 1993, 1994; Yang and Yeh 1994; McPherson and Wilson 1995; Liang and Wilson 2002; Wilson and Wheeler 2005; Emeljanov 2009), none of which are from South America.

*T. longula* is a planthopper that breeds on water hyacinth, *Eichhornia crassipes* (Martius) Solms-Laubach (Commelinales: Pontederiaceae), in northeastern Argentina on the Paraguay River basin and upper Amazon River. *E. crassipes* is under study as a candidate for water hyacinth biocontrol (Hernández et al. 2011a, b). The adults are characterized by the uniform green coloration with a pair of lateral small dark spots on the mesonotum, vertex subquadrate with a closed triangular facet well defined on apex, and a long anal segment. Males have suboval parameres with subequal-sized teeth shorter than the anal segment, and the tubular aedeagus with a pair of ventral spinose processes is recurved upward. The females have a straight and long gonapophysis VIII with a row of nine bifurcate teeth on the apical fourth (Remes Lenicov and Hernández 2010). Biological data based on laboratory rearing and field observations showed that *T. longula* causes serious feeding damage and is host specific on water hyacinth. In feeding tests on another species of Pontederiaceae, *Pontederia cordata* L., *T. longula* survived to fourth instar and were only able to develop to adults on water hyacinth. Nymphs and adults are sap feeders, mainly phloem feeders, producing true salivary sheaths (Hernández et al. 2011a, b; Cabrera et al. unpublished). The current study provides a description of the immature stages of *T*. (*C*) *longula* and includes a key for identification. Some biological information on behavior and development is also given.

## Materials and Methods

### Collection site and biological observation

The descriptions and biological observations of immature stages were done on specimens collected in Herradura, Formosa Province (26° 29′ 28″ S; 58° 18′ 37″ W) and Chaco Province (27° 26′ 28″ S; 58° 53′38″ W), Argentina. Water hyacinth petioles with *T. longula* clutches from the field were incubated in the laboratory (25 ± 1° C; 70 ± 10% RH, 14:10 L:D) in groups of 10–20 petioles in 40 × 27 × 8 cm plastic trays lined with moist tissue paper and covered with adhesive film. To obtain the specimens of different instars, the newly emerged instars were collected with aspirators, and five of them on one excised water hyacinth leaf were placed into a cylindrical plastic container (7 cm wide × 5 cm tall) with perforated lids. The container was lined with moist tissue paper and maintained in the same conditions mentioned above. Six containers were filled. The leaves were changed periodically before visible decay appeared and inspected daily to detect molts. Six individuals of each instar were fixed in 70% ethanol.

### Morphological Studies

The living specimens were anesthetized with 95% ethyl ether to maintain coloration, cleared in 10% cold potassium hydroxide (KOH), and mounted in Faure liquid for microscopic examination. The fifth instar nymph is described in detail; only the major differences are highlighted for earlier stages. Measurements are from 10 specimens of each sex and given in millimeters ± SD. The dimensions are expressed as total body length (from the tip of the vertex to the tip of abdomen), maximum body width (at the level of the metanotum), thoracic length (from the anterior margin of the pronotum to the posterior margin of the metanotum along the midline), and anterior wingpad length (from the anterior margin of mesonotum to the tip of wingpad along the ventro-lateral margin). Other measurements are relative. The nomenclature for the arrangement of pits follows Vilbaste (1968). Drawings were made with camera lucida on an Iroscope stereomicroscope.

**Specimens Examined.** ARGENTINA: Formosa, Province, Herradura, (26° 30′ 59″ S; 58° 16′ 54″ W), 11-II and 1-XII-2004, 10 nymphs V, five male and five female, 10 nymph IV, 10 nymphs III, 10, nymphs II and 10 nymphs I, on *Eichhornia crassipes* (Martius) Solms-Laubach, Hernandez and Sacco col. PERU, Montoya Cocha. (4° 31′ 00″ S; 73° 32′ 23″ W), 3-V-1999, eight nymphs V on *E. crassipes*, H. Cordo col. Other specimens examined: reared at SABCL on *E. crassipes*, Hurlingham, Buenos Aires, 20-VI-2007, from Palo Santo, Formosa (25° 33′ 42″ S; 59° 19′ 10.6″ W) on *E. crassipes*, Hernández and Sacco, col.

## Results

**Figure 1.**
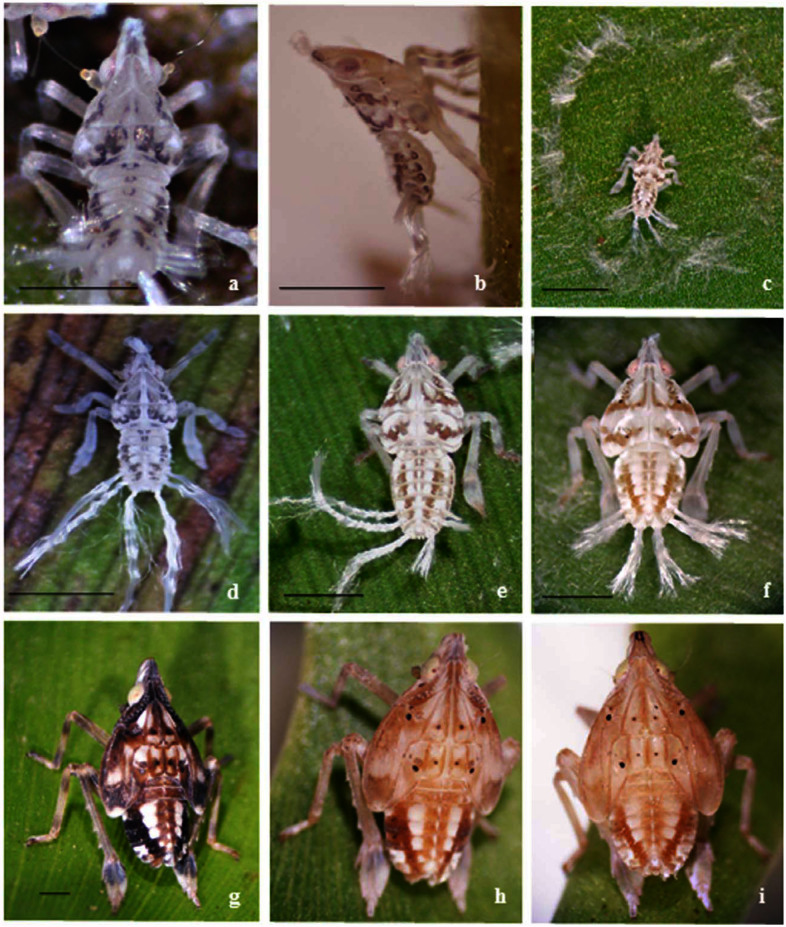
Nymphal instars of *Taosa* (*C*.) *longula*. (a) first instar, dorsal; (b) first instar, lateral; (c) second instar in the waxy hair circle; (d) second instar; (e) third instar; (f) fourth instar; (g, h, i) fifth instar, different tones. Scale bar: I mm. High quality figures are available online.

**Figure 2.**
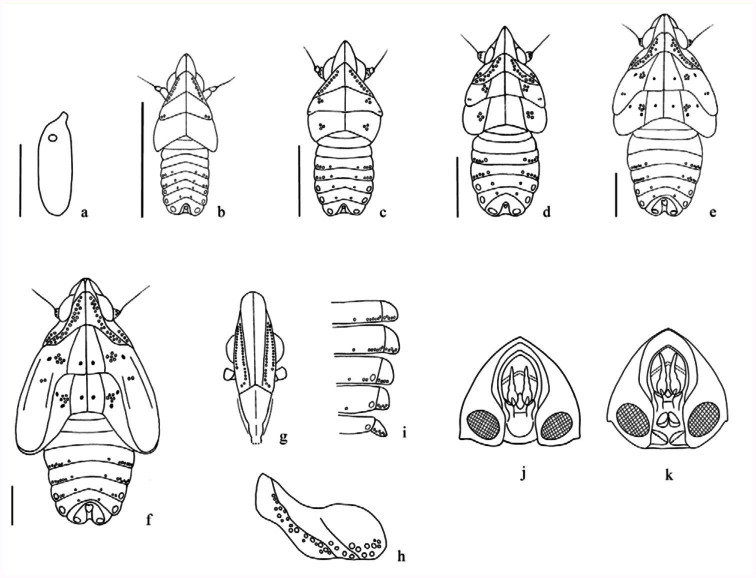
Egg and nymphal instars of *Taosa* (*C*.*) longula*. (a) egg; (b) first instar; (c) second instar; (d) third instar; (e) fourth instar; (f to k) fifth instar; (g) head; (h) right pronotal plate, flat surface; (i) abdominal tergites and pleurites IV–VIII, flat surface; (j) male abdominal segment VIII–X, caudal view; (k) female abdominal segment VIII–X, caudal view. Scale bar: 1 mm. High quality figures are available online.

**Figure 3.**
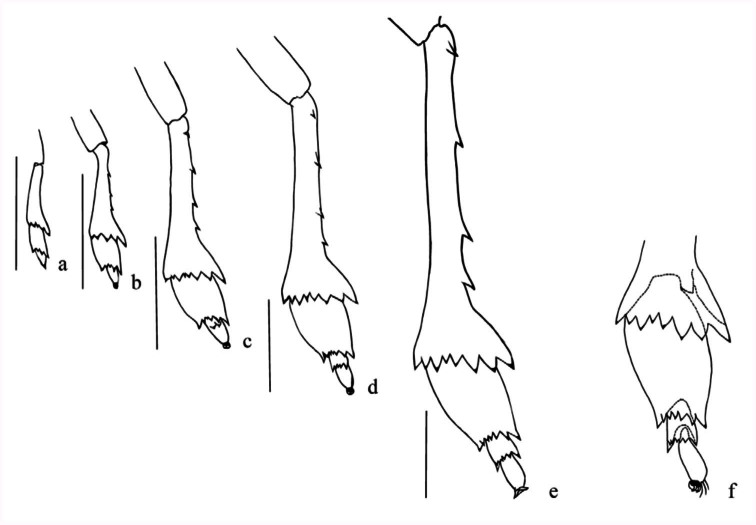
Metatibia and metatarsi of *Taosa* (*C*.) *longula*. (a) first instar; (b) second instar; (c) third instar; (d) fourth instar; (e) fifth instar; (f) apex of metatibia and tarsal segment, ventral. High quality figures are available online.

**Immature stages of *Taosa longula* Remes Lenicov, 2010**
**Eggs.** Length: 1.4 (± 0.01), width: 0.5 (± 0.01).
  Eggs milky white, ellipsoidal with cephalic apex truncated; ventral surface slightly flat and dorsal convex (Figure 2a). Chorion translucent, with a polygonal chorionic microsculpturing that covers the apical fourth and vanishes to the middle with open polygons.
**Fifth-instar nymph** (Figures 1g, h, i; Figures 2f, g, h, i, j, k; Figures 3e, f). Length: 6.24 (± 0.37); width: 3.58 (± 0.04); thoracic length: 3.54 (± 0.08); anterior wingpad length: 2.85 (± 0.14).
  Varied coloration. Eyes red; general coloration pale-brown or whitish to pale-yellowish with dark-brown marks on dorsum of head, thorax, and abdomen, which contrast with the whitish ground coloration. One set of three longitudinal brown stripes, medial and sub laterals, along the head and the pronotal and mesonotal plates posteriorly joined in a broad transversal stripe that covers anterior 2/3 of mesonotum; a second transversal brown stripe, “w” like, on posterior ⅔ of metanotum except the apex of mesonotal and metanotal wing pad. Abdomen with four longitudinal stripes, submedial and sublateral, with sublaterals converging at mid-dorsal line of urotergite VII. Both coloration patterns, uniform green or striped, have two and three small black spots on each one of the mesonotal and metanotal plates, respectively. Femora, pro, meso, and metatibiae each with about two ring-like faint stripes, metatarsi, brownish at basal ⅓. Head protruding far beyond anterior margin of eyes. Vertex 1.3 times longer in midline than it is wide at base.
  Frons about two times longer in middle line than it is wide at widest part (widest near frontoclypeal suture); submedian carinae almost reaching frontoclypeal suture converging from base to apex ending quite close to each other; median carinae distinct along frontal length. Each side of frons with ca. 38 pits; 19–22 regularly distributed on external side of each sub median carina, the apical one a bit separated from the carina, and 16–19 on inner side of lateral carinae from base to beyond the anterior margin of the eyes, at the level of the ocelli (Figure 2g). Anteclypeous longer than it is wide at base (1.7:1), distinctly 3-carinate along its length; fronto clypeal suture inverted V shaped. Rostrum 4-segmented, exceeding metacoxae, segment I obscured by postclypeus; subapical segment 1.2 times longer than the apical. Antennae 3-segmented: scape short, ring-like; pedicel sub cylindrical ca. four times the length of the scape, with about 25–28 pits on the apical half; flagellum whip-like distally, bulbous at base.
  Thoracic nota divided by longitudinal mid-dorsal line into three pairs of plates. Pronotal plates strongly projected anteriorly, reaching about the anterior margin of eyes, each one with a long sinuous lateral carina in median ⅕ extending up to posterior margin, and a single straight humeral carina in median ⅓ at posterior margin. Each plate with ca. 32 pits; 18–20 dorsal pits irregularly placed in two rows just posterior to lateral carina; four pits lined between lateral and humeral carina, and 8–10 pits irregularly placed on lateral-posterior angle, not visible in dorsal view (Figure 2h). Mesonotal median length 2 times that of the pronotum, each plate with distinctive lateral carina originating on anterior margin in median ⅕ extending posterolaterally, reaching posterior margin with a cluster of five to seven pits just lateral to carina, and two small pits on wingpad internal to the median provein (between costal-medial and cubito-anal sections (as Trueman, 1989)); wingpads lobate, extending to apex of metanotal winpads. Metanotal median length around 0.5 times that of the mesonotum; each plate with a distinct subparallel longitudinal carina in median ⅓, with a cluster of five to eight pits just lateral to carina; wingpads broadly lobate, extending laterally near to posterior margin of urotergito III. Pro- and mesocoxae slender, ridged; mesocoxae with process on both sides on basoventral angles. Pro and mesofemora at both ventral margins indistinctly dentate. Pro and mesotarsi each with two tarsomeres; tarsomere I wedge-shape, tarsomere II, subcylindrical, three times longer than I on plantar surface. Metatrochanters each with a row of 11–13 interlocking flattened folds medially. Metatibia (Figure 3e) each with four lateral teeth and a transverse apical row of nine spines (eight on plantar surface). Metatarsi (Figure 3f) with three tarsomeres; metatarsomere I with both sides extremely blade-shaped expanded, in dorsal view median line elevated and ridged, with transverse apical row of seven spines on plantar surface, length two times the II + III; metatarsomere II subtriangular, with apical row of six to seven spines on plantar surface (generally seven); metatarsomere III, subcylindrical, length two times the tarsomere II. Spinal formula of hind leg 9-7-7. All legs with terminal pair of curved claws and a membranous median pulvillus.
  Abdomen nine-segmented, widest across segment III. Tergites VII-IX telescoped anteriorly, segments VI-VIII each with oval dark cream wax pads at caudolateral portion of each tergite-bearing minute pore. Segment IX slightly sclerotized, small, ring-like and elongate vertically, in dorsal view sunk into emargination of eighth segment, locked anal segment; with one pit on each side placed on a sclerotized protrusion below base of anal combs, which are lobe-like membranous dorsally directed processes and slightly wider at base. Anal combs length 5x its wide at base, slightly longer in female nymph (fig.2j, k). Abdominal tergite IV-V each with (1 + 6), VI each with (1 + 2), VII each with one, VIII without pits and IX each with one pit on either side of midline; pleurites IV-V each with five, VI each with four, VII each with five to six, and VIII each with five to eight pits (Figure 2i).
**Fourth-instar nymph** (Figure 1f; 2e; 3d). Length: 4.64 (± 0.16); width: 2.32 (± 0.08); thoracic length: 1.96 (± 0.05).
  Ground color whitish with similar coloration pattern, but mesonotal and metanotal transversal dark stripes and abdominal marks lighter. Median and lateral pair of small black spots on each thoracic plates similar to the oldest instar. Vertex more than 1.3 times as long as it is wide at base. Each side of frons with about 34 pits, around 19 regularly distributed on external side of each submedian carinae and around 15 on inner side of lateral carinae from base to beyond the anterior margin of the eyes. Rostrum exceeding metacoxae, segment I obscured by postclypeus; subapical segment 1.2 times longer than the apical. Antennae three-segmented: pedicel subcylindrical, with about 11–14 pits on apical half. Pronotal plates projected toward vertex at level of about ⅓ anterior margin of the eyes; each with around 23 pits; 11–13 dorsal pits arranged in two irregular rows just posterior to lateral carina; four pits lined between lateral and humeral carina and six irregularly placed on laterad-posterior angle. Mesonotal plate with a cluster of four to five pits just lateral to carina and two small pits on wingpad internal to the median provein; wingpads lobate, surpassing half of the length of metanotal winpads. Metanotal plate with a cluster of four to six pits just in medium lateral to carina; wingpads broadly lobate, extending laterally at level of anterior margin of urotergite III. Metatrochanters each with a row of nine to 11 interlocking flattened folds medially. Metatibia (Figure 3d), each with an apical row of nine spines (eight on plantar surface). Metatarsomere I with apical row of six to seven spines on plantar surface (generally seven); metatarsomere II with apical row of four to five spines on plantar surface (generally five). Spinal formula of hind leg 97-5. Metatarsomere I length, two times that of II plus III; metatarsomere II and III subequal. Abdominal tergite IV–V each with (1 + 5), VI–VII each with one, VIII without pits and IX each with one pit on either side of midline; pleurites IV each with six, V–VII each with four, VIII each with five to six pits.
**Third-instar nymph** (Figures 1e; 2d; 3c). Length: 3.18 (± 0.19); width: 1.5 (± 0.01); thoracic length: 1.54 (± 0.23).
  Ground color whitish with the transversal thoracic stripes, longitudinal sublateral on urotergites and tarsites light-brown. Vertex more than 1.3 times as long as it is wide at base. Each side of frons with about 28±32 pits, around 19 regularly distributed on external side of each submedian carina and around 13 on inner side of lateral carina from base to middle eyes. Rostrum exceeding metacoxae, apical, and subapical segment subequal. Antennae with pedicel subcylindrical with about five to six on apical half. Pronotal plate margin slightly projected toward vertex (at level of ⅓ posterior margin of the eyes); each with around 22 pits; 11–14 dorsal pits irregularly arranged in one row just posterior to lateral carina; four pits lined between lateral and humeral carina and three to four at laterad posterior angle. Mesonotal and metanotal plates slightly rounded laterally, subequal in median and lateral length; mesonotal plate with a cluster of four to five pits lateral to carina and two near the external margin on wingpad. Mesonotal wingpads covering ¼ of metanotal ones. Metanotal plate with a cluster of four to five pits just lateral to carina; wingpads extending laterally at level of anterior margin of urotergito II. Metatrochanter each with a row of about 11–13 interlocking flattened folds medially. Metatibia (Figure 3c) each with apical row of nine spines (eight on plantar surface). Metatarsomere I with an apical row of five to six spines on plantar surface, metatarsomere II, with two weakly developed ventral spines near middle of plantar surface. Spinal formula of hind leg 9-6. Abdominal tergite IV–V each with (1 + 4), VI–VII each with one, VIII without pits and IX each with one pit on either side of midline; pleurites IV-V each with four, VI-VIII each with three pits.
**Second-instar nymph** (Figures 1c, d; 2c; 3b). Length: 2.37 (± 0.29); width: 0.97 (± 0.05); thoracic lenght: 0.87 (± 0.05).
  Ground color whitish with similar pattern to third instar, but lighter. Vertex as long as it is wide at base. Each side of frons with 22 pits, around 12 regularly distributed on external side of each sub median carina and around 10 on inner side of lateral carina from base to middle eyes. Rostrum apical and sub apical segments sub equal. Antennae with pedicel sub cylindrical bearing three apical pits. Pronotal plates margin slightly projected toward vertex (at level of ⅓ posterior margin of the eyes); each one with around 16 pits; nine to 10 dorsal pits, irregularly arranged in one row just posterior to lateral carina; four pits lined between lateral and humeral carina and two at ventral margin. Mesonotal and metanotal plates sub equal in shape and length; mesonotal ones each with a cluster of three pits lateral to carina and two near of the external angle; the metanotal each with a cluster of three to four pits just lateral to carina; wingpads extending to anterior margin of urotergite II. Metatrochanter each with a row of about nine to 12 interlocking flattened folds medially. Metatibia (Figure 3b) each with three lateral teeth and an apical row of six spines (five on plantar surface). Metatarsomere I with an apical row of four to five spines on plantar surface. Spinal formula of hind leg 6-5. Abdominal tergite IV–V each with (1 + 3); VI–-VII each with one; VIII without pits and IX each with one pit on either side of midline; pleurites IV–VIII each with two pits respectively.
**First-instar nymph** (Figure 1a, b; 2b; 3a). Length: 1.40 (± 0.10); width: 0.6 (± 0.01); thoracic length: 0.57 (± 0.05).
  Ground color whitish with similar pattern to second instar but lighter. Head in dorsal view, protruding out at the anterior margin of the eyes as far as it is wide at its base. Vertex as long as it is wide at base. Each side of frons with 22 pits; around 12 regularly distributed on external side of each sub median carina and around 10 pits on inner side of lateral carina from base to middle of the eyes. Antennae with pedicel without sensory pits; segment III bulbous basally bearing a plaque organ. Pronotal plates margin projected toward vertex at level of anterior margin of the eyes; each one with about eight pits regularly arranged in one row just posterior to lateral carina. Notal plates subtrapezoidal, pronotal ones with prominent and divergent lateral carina. Posterior margin of mesonotal plates straight, metanotal widely concave; mesonotal ones each with a cluster of two pits near the external margin; the metanotum without distinguishable pits. Metatrochanter each with a row of about 11–12 interlocking flattened folds medially. Metatibia (Figure 3a) each without lateral teeth and an apical row of five spines on plantar surface, the dorsal ones barely developed; metatarsomere I with an apical row of four spines on plantar surface. Spinal formula of hind leg 5-4. Abdominal tergite IV–V each with (1 +2); VI–VII each with one; VIII without pits and IX each with one pit on either side of midline; pleurites IV–VIII each with one pit, respectively.

**Key to nymphal instars of *T*. (*C*.) *longula***
1. Metatarsi three-segmented 2
— Metatarsi two-segmented 3
2. Mesonotal wing pads reaching nearly to apex of metanotal ones. Metatarsomere I and II bearing ventral apical transverse row of 7 black-tipped spines Fifth-instar nymph
— Mesonotal wing pads extending laterally more than half of the metanotal ones. Metatarsomere I bearing ventral apical transverse row of 6–7 black-tipped spines; metatarsomere II with 4–5 ones Fourth- instar nymph
3 .Metatarsomere II, with 2–3 weakly developed ventral spines near middle of plantar surface Third-instar nymph
— Metatarsomere II without ventral spines in the middle of tarsomere 4
4. Metatibia with 3 lateral spines; antennal pedicel with apical sensory pits Second-instar nymph
— Metatibia without lateral spines; antennal pedicel without sensory pits First-instar nymph

## Biological information

All stages of *T. longula* were observed breeding exclusively on water hyacinth, *E. crassipes*. This aquatic plant is native to South America, and prospers in tropical to subtropical river basins. Currently it has spread in tropical and warm regions all over the world, and has become one of the most serious weeds (Hernández et al. 2011b). The plants grow in dense mats (mean 60 cm tall) over the water, producing a micro-environment under the canopy suitable for the establishment of planthopper populations. Female *T. longula* lay clutches of eggs in the middle part of the petioles. The young nymphs remain together, most often in the abaxial lamina, while the remainders are distributed from the adaxial lamina to the mid-petiole. They spread their waxy filaments around the feeding area (Figure 1c). Older instars and adults become more solitary and move daily from the laminas to the base of the plant during the hottest period of the day. In Chaco Province (27° 26′ 28″ S; 58° 53′ 38″ W), the southern distribution observed, *T. longula* was observed year round, albeit with a clear decline in the number of adults and nymphs during early winter. They can overwinter as eggs, and the nymphs emerge at the end of the winter. A group of clutches collected at the end of autumn remained unhatched for nearly 75 days in an unheated greenhouse. The mean developmental time of the nymphal instars (N1 to 5), feeding on water hyacinth at 25° C, was 33.75 days from hatching to adult, distributed in N1: 6.1days (± 0.27); N2: 5.4 days (± 0.54); N3: 5.1days (± 0.9); N4: 7.7 days (± 0.31); N5: 14.7 days (± 1.72). The durations should be longer under natural conditions.

## Discussion

Immature specimens collected in the field had greater variation in the pattern of coloration than in morphology. Coloration pattern and the morphology of the head (shape, carination of head, and number of frontal sensory pits) are the most useful diagnostic features. Fifth nymphal instars of *T*. (*C*.) *longula* may be easily separated from the only congener previously described, *T. inexacta* (according to the description of Yang and Yeh 1994), by the brown marked pattern coloration, shorter vertex, and the distinguishable median carina along the frons.
